# Cell-targeting antibodies in immunity to Ebola

**DOI:** 10.1093/femspd/ftw021

**Published:** 2016-03-21

**Authors:** Alan Schmaljohn, George K. Lewis

**Affiliations:** 1 Microbiology & Immunology, University of Maryland School of Medicine, USA; 2 Institute of Human Virology, University of Maryland School of Medicine, 725 W. Lombard St., Baltimore, Maryland, 21201, USA

**Keywords:** Ebola, antibody, vaccine, therapy, Fc, virus

## Abstract

As the 2014–15 Ebola virus epidemic in West Africa evolved from emergency to lesson, developers of both vaccines and therapeutic antibodies were left with the puzzlement of what kinds of anti-Ebola antibodies are predictably desirable in treating the afflicted, and what antibodies might account for the specific and lasting protection elicited by the more effective vaccines. The facile answer in virology is that neutralizing antibody (NAb) is desired and required. However, with Ebola and other filoviruses (as with many prior viral examples), there are multiple discordances in which neutralizing antibodies fail to protect animals, and others in which antibody-mediated protection is observed in the absence of measured virus neutralization. Explanation presumably resides in the protective role of antibodies that bind and functionally ‘target’ virus-infected cells, here called ‘cell-targeting antibody’, or CTAb. To be clear, many NAbs are also CTAbs, and in the case of Ebola the great majority of NAbs are likely CTAbs. Isotype, glycosylation, and other features of CTAbs are likely crucial in their capacity to mediate protection. Overall, results and analysis invite an increasingly complex view of antibody-mediated immunity to enveloped viruses.

## Abstract (precis)

Immunity to Ebola and other enveloped viruses often involves cell-targeting antibodies, which mark virus-infected cells for Fc-dependent immune attack and entail previously underappreciated complexities of Fc type and Fc–FcR interaction.

## PROTECTIVE CTABS, NEUTRALIZING AND NON-NEUTRALIZING

**Figure 1.**
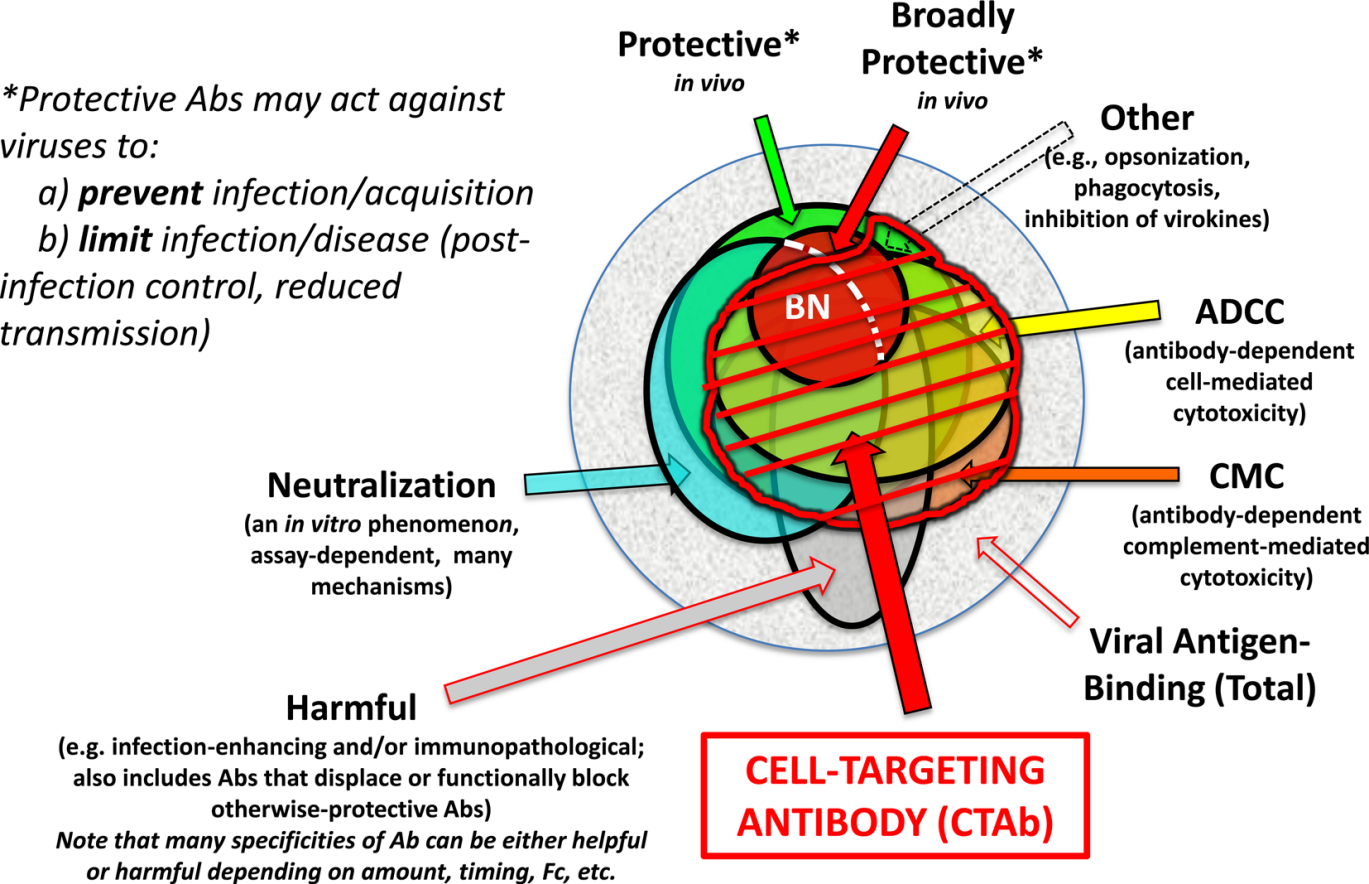
Euler diagram of CTAb in the context of functional antibodies to an enveloped virus. **(a)** Within the total population virus-reactive antibodies that arise in an individual host, some can be demonstrated to protect against disease caused by the homologous virus, and a subset of these are broadly protective against related viruses. (**b)** Among the antibodies that can be defined as virus-neutralizing *in vitro* through any of several mechanisms and assays (see text), some are broadly neutralizing (BN), many but not all are protective *in vivo*, and neutralizing antibodies do not account for all protective antibodies. (**c**) Antibodies responsible for ADCC and complement-mediated cytolysis (CMC) form distinct but highly overlapping sets, and depending upon the antigen (as well as Ig isotype and other factors) may include a high proportion of neutralizing antibodies. ‘Other’ protective antibodies are described elsewhere (Schmaljohn 2013) (**d**) Antibodies of all types may occasionally be harmful to the host through mechanisms that include autoimmunity, antibody-dependent enhancement of viral infection, increased virus-specific immunopathology, displacing or functionally blocking otherwise-protective antibodies or creating immunosuppressive immune complexes. Some antibodies may be categorized as both protective and harmful, with outcome depending on such things as antibody concentration, timing of antibody arrival relative to viral load and host variations in inflammatory response. It can further be inferred that single antibody specificity (as defined by clonal lineage and paratope) may be protective, harmful or impotent depending upon Ig isotype and its consequences. (**e)** CTAb, often polyfunctional, are those that bind viral antigen on virus-infected (and sometimes uninfected but virus-sensitized) cells, potentially marking cells for damage or destruction by FcR-bearing cells before peak viral production by the antigen-bearing cells. Antibody-mediated protection *in vivo* is very often dependent upon Fc–FcR interactions regardless of whether antibodies are also categorized as neutralizing. Opsonization facilitated by antibodies against virion surfaces may play a role *in vivo*, but the larger body of virological evidence points toward CTAb mediating protection in concert with FcR-bearing cells of various types.

In reporting findings with monoclonal antibodies (MAbs) some 34 years ago (Schmaljohn *et al.*^1982^), the term ‘non-neutralizing’ seemed helpful to emphasize that NAbs are not solely responsible for antibody-mediated protection against viruses, and that other Abs (lacking in demonstrable neutralizing activity) are also important for many viruses. Today, the distinction between neutralizing and non-neutralizing Abs serves mostly to create a false dichotomy in which devout adherents of a notional *in vitro* phenomenon called ‘neutralization’ endeavor to dismiss CTAbs. The time has surely arrived to retire the term ‘non-neutralizing antibodies’ as a negative descriptor, which for the sake of precise language must be regularly distinguished as either protective or non-protective. There is common ground in the data. NAbs are important, and so are CTAbs, and foremost in this regard, many NAbs are CTAbs (Schmaljohn 2013). If the term CTAb describes an expansive set of antibodies that generally includes NAbs (Fig 1), does it matter if research efforts (and funding) revolve almost exclusively around NAbs? Yes, insofar as NAbs are potentially polyfunctional *in vivo*, either preventing viral entry into cells or alternatively acting at a later step in viral replication, perhaps in concert with Fc receptor-bearing cells or complement. For the latter mechanisms, the Fc portion of the Ab molecule may be decisive in the quality of antiviral effect observed *in vivo*, partially or wholly independent of the neutralization activity observed *in vitro* (Hessell *et al.*^2007^; Boesch, Brown and Ackerman 2015; Chung *et al.*^2015^). Even more obviously—and where unhelpful battle lines are sometimes drawn—there exist antibodies that protect wholly as CTAbs despite lacking neutralization activity and sometimes lacking even the capacity to bind virion surfaces (Schmaljohn 2013).

## COMPLEXITY IS NOT ONLY FOR T CELLS

Evolution—through the agency of a complex adaptive immune system—is preoccupied with successful antiviral defenses, not with our semantics. Thus, when ‘simple’ antibody paratope-driven neutralization is not the only or even the principal means of resistance to a given virus (here, Ebola), rational design (prediction, interpretation) and improvement of vaccines and therapies demands that we consider a somewhat bewildering array of complexities (Plotkin 2013; Excler *et al.*^2014^; Bournazos and Ravetch 2015; Bruhns and Jönsson 2015). Fc and Fc receptors (FcR) come to the forefront in an elegant coordination between flexible immunoglobulin molecules and an array of FcR-bearing cells, together bent on combat against cells that have viral antigens on their surfaces (Casadevall and Pirofski 2012; DiLillo *et al.*^2014^; Lewis 2014; Wang *et al.*^2015^). The capacity of the larger immune system to create and sustain not only the correct paratopes but the correctly matched Fc for adequate antiviral activity is a remarkable feat, and is not yet understood to a degree that allows rational forecast and manipulation of outcomes. The clinically relevant problem, then, is more than one of statistical correlates between assay and effectiveness, but of substantial understandings that can guide progress on a wider scale. How is protection against severe viral disease influenced by antibody quantity, specificity, affinity (for a particular conformational state of cognate antigen), isotype, immunoglobulin heavy chain mutations and post-translational modifications, biodistribution, half-life, memory, and host FcR polymorphisms? Where do prozone (Lewis 2013) and negative feedback from immune complexes (Yamada *et al.*^2015^) enter the equation? Beyond empiricism, how do we conspire to shape vaccine-induced immune response not only in terms of specificity, but other identified and desirable traits of antibodies? We can safely presuppose that the entire immune system—from antigen presentation to T cells—influences and guides the repertoire of protective antibodies. To acknowledge these complexities is to confront the current state of immunological knowledge, a matter of both humility and opportunity.

## EBOLA VIRUS DISEASE (*EVD) AND ANTIBODIES, A BRIEF HISTORICAL REVIEW*

As the recent Ebola epidemic in West Africa unfolded, the first report most people heard of a possible treatment was something described in popular press as a ‘miracle drug’, an antibody cocktail in ongoing development under the name of ZMapp. For Filovirus cognoscente, this particular cocktail was part of a long and unfinished search for both therapeutic antibodies and an understanding of the immune responses wished for in response to vaccines (Wilson *et al.*^2000^; Qiu *et al.*^2012^; Pettitt *et al.*^2013^; Murin *et al.*^2014^; Hiatt *et al.*^2015^). Earlier, clinical studies with convalescent plasma from patients recovered from either Marburg or Ebola viruses were anecdotal and inconclusive in terms of therapy against the respective viruses in newly infected patients (Mupapa *et al.*^1999^), and a report from the most recent epidemic affirmed the absence of significant efficacy of convalescent plasma (van Griensven *et al.*^2016^). Similarly, early experiments with immune (anti-Ebola) sera transferred into non-human primates (NHP; subsequently infected with a lethal strain of Ebola Zaire) were initially variable and ultimately inconclusive, but were more encouraging when high doses of concentrated IgG were given (Dye *et al.*^2012^). Meanwhile, early studies with Marburg virus demonstrated three essential findings: (1) convalescent guinea pig serum prevented lethal disease in guinea pigs; (2) mouse monoclonal antibodies (MAbs) against Marburg glycoprotein (GP) showed initial promise in protecting guinea pigs; and 3) in non-human primates, GP (the only known target for either NAbs or CTAbs) was a necessary and sufficient component of a successful vaccine (Hevey *et al.*^1997^, ^1998^; Hevey, Negley and Schmaljohn 2003). For Ebola, MAbs were produced in mice and tested for several activities including *in vitro* neutralization, breadth and specificity of reactivity and protection of mice against a mouse-adapted variant of Ebola virus. The conclusions were that both neutralizing MAbs and non-neutralizing MAbs were protective against EVD in mice (Wilson *et al.*^2000^; Qiu *et al.*^2012^), and since all protective MAbs were directed against GP, all were presumed or directly shown to be CTAbs. With Ebola as with many other viruses (Schmaljohn 2013), there were hints of the importance of antibody isotype in protection, and indications of antibody efficacy in post-infection immunotherapy. The differences among the many studies could be reconciled easily enough by hypothesizing that antibody-mediated protection against EVD is indeed important in immunity, but that convalescent plasma contains too little of the most desirable antibodies (or antibody combinations) to be effective.

Antibody enthusiasts paused briefly to reconsider the path forward when a highly potent neutralizing MAb of human IgG1 type, obtained from an EVD-convalescent individual, proved to be protective in guinea pigs but failed to prevent or substantially diminish EVD in NHP, the more sensitive and relevant model of EVD (Oswald *et al.*^2007^). In an effort to maximize therapeutic efficacy in NHP—and frankly save both time and NHP—two separate groups pivoted toward antibody cocktails consisting of mouse MAbs converted to human IgG1. Success was encouraging but incomplete, and a pragmatic alliance was formed to select the most promising individual MAbs from both laboratories (choices may have reflected a bias toward NAbs, but no bias against CTAbs), and to produce three MAbs as human IgG1 in a *Nicotiana* (tobacco plant) system designed for scale-up and for exquisite control of antibody glycosylation (Zeitlin *et al.*^2011^; Hiatt *et al.*^2014^). In preclinical studies, the new cocktail (ZMapp) was astonishingly successful in NHP (Qiu *et al.*^2014^), especially given historical difficulties of immunotherapy or drug therapies against EVD in NHP. As the epidemic unfolded in West Africa, ZMapp—still available in only limited quantities—was offered for emergency compassionate use for a few patients. Due to small numbers in a necessarily uncontrolled study, analysis of ZMapp efficacy in immunotherapy against human EVD remained incomplete or anecdotal, perhaps a miracle drug of sorts, perhaps not. This body of work was recently reviewed (Hiatt *et al.*^2015^) with associated references.

## CTAB AND ADCC

In the case of Ebola and most other viruses for which CTAb are implicated, there is a notable paucity of direct and convincing mechanistic evidence about how CTAb may exert antiviral effects. If neutralization is the first and most facile explanation for the antiviral effects of antibody, then antibody-dependent cell-mediated cytotoxicity (ADCC) is the second. Before considering ADCC, it is important to remind that neutralization is itself a polythetic and operational term for which neutralizing antibodies may share some but not all characteristics, and viral neutralization is only defined by the particular assay in use for a given virus (Schmaljohn 2013). Consequently, it is less troubling to assert that ADCC too has no single meaning, and that different assays provide different interpretations of the biological phenomena that give rise to protection by CTAb (Golay and Introna 2012). From the earliest observations of protection by non-neutralizing antibodies, most of us sidestepped the issue, generally using complement-mediated lysis of cells (more recently, flow cytometry) to demonstrate whether an antibody's cognate antigen were available on virus-infected cell surfaces, but not asserting a definitive mechanism of protection *in vivo*. Also, from the earliest MAb reports (and understood as such by many prior virologists), NAbs were observed to be implicitly polyfunctional, exerting antiviral effects *in vivo* not only by preventing cell infection but possibly by opsonization and aggregation of virions, as well as by Fc-dependent activities at the cellular level such as ADCC (Schmaljohn *et al.*^1982^; Schmaljohn, Kokubun and Cole 1983; Schmaljohn 2013).

To say that ADCC assays are rife with both complexity and misunderstanding may be an understatement: classical assays measured cytolysis of target cells via chromium-release; some newer and high-throughput assays may measure triggering of a particular kind of effector cell (e.g. NK cell) or cell line; other rapid fluorometric assays were originally thought to measure cytotoxicity, but may in fact measure trogocytosis (antibody-facilitated acquisition of target cell membrane by effector cell) (Kramski *et al.*^2012^, ^2013^; Hu *et al.*^2014^). Other assays measure phagocytosis (Ackerman *et al.*^2013^). The antibodies and effector cells of some animal species, including mice, guinea pigs and even NHP, have proven intractable for some of these assays, arguably providing legitimate excuse for the paucity of ADCC data with Ebola virus (Warfield *et al.*^2007^). ADCC assays in the human antibody-effector system are generally far more pliable, with the most abundant and compelling data coming from anti-tumor CTAbs (Golay and Introna 2012; Modjtahedi, Ali and Essapen 2012) and also from HIV research, where interest in ADCC was greatly piqued by a moderately successful vaccine study (called RV144) in which antibodies other than typical neutralizing antibodies were incriminated in protection (Haynes *et al.*^2012^). For HIV, a systematic and coordinated search was begun for assays—including various and sometimes discordant ADCC assays—that aligned with the observed efficacy of vaccine. Much of this research has been reviewed recently (Ackerman, Dugast and Alter 2012; Lewis 2013, 2014; Pincetic *et al.*^2014^) and illustrates that ADCC is more complex than most had imagined. As a final note on the complexities, the prior review (Schmaljohn 2013) also described a few circumstances in which antibodies may protect neither by neutralization nor by Fc-dependent mechanisms, but by such paratope-focused activities as inhibition of soluble viral virulence-enhancing proteins, inhibition of essential viral envelope cleavage or inhibition of viral release.

## EBOLA, FC AND SOME CLUES IN THE DETAILS

An emerging body of research points toward the immunoglobulin molecule as a dynamic entity with reciprocal allosteric effects between paratope and Fc. The contortions of the Ig molecule, and the biological consequences of same, may vary greatly with heavy chain isotype and/or post-translational modifications, especially near the hinge region of the molecule (Casadevall and Pirofski 2012; DiLillo *et al.*^2014^). In this regard, it is noteworthy that ZMapp was designed and produced with a particular kind of glycosylation important in human IgG1 dynamism (Zeitlin *et al.*^2011^; Hiatt *et al.*^2014^, ^2015^). Intermediate studies on the pathway to ZMapp, more empirical than mechanistic, had suggested this modification to be useful in enhancing an antibody's capacity to protect NHP against EVD. Neutralization (*in vitro*) was unchanged, and the suggestion was that the biological effect of CTAb was improved *(ibid)*. Cumulatively, these observations cry out for verification, extension and deep analysis: the implications are too important to ignore. Moreover, they add caution to a casual efficiency-driven change in the way ZMapp antibodies are produced, and invite head-to-head NHP protection comparisons among antibodies differing in only subtle ways in their Fc regions.

## OTHER SUPPORTIVE EVIDENCE FOR CTAB

Many lines of evidence have emerged over the last four decades supporting the importance of CTAb in antiviral immunity, and these were reviewed recently (Schmaljohn 2013). Three of the more recent studies are particularly illustrative. First example: West Nile virus (WNV), a flavivirus, makes a non-structural glycoprotein (NS1) that is found on infected-cell surfaces but not on virions, and anti-NS1 antibodies do not neutralize WNV in any *in vitro* assay described. Nevertheless, anti-NS1 MAbs prevent lethal WNV disease (manifested as encephalitis) in mice. Ig isotype appeared to be important in protection, and most significantly the protection was ablated in only a subset of knockout mice: those lacking FcRgIII, a protection that was associated with the capacity of macrophages to phagocytose WNV-infected cells in the presence of anti-NS1 MAb (Chung *et al.*^2007^). The role of ADCC was not directly assessed. Parenthetically, among Flaviviruses, protection by anti-NS1 was first observed with yellow fever virus, YFV (Schlesinger, Brandriss and Walsh 1985) and absence of anti-NS1 antibody could hypothetically account for underperformance of a live-attenuated dengue vaccine that elicited neutralizing antibodies against the dengue E protein but no antibodies to homologous NS1, which in the chimeric vaccine virus was derived from the dissimilar YFV (Sabchareon *et al.*^2012^); an alternative hypothesis is that the classical neutralization assay is flawed because it does not adequately reflect cell tropisms *in vivo* (Tsai *et al.*^2015^). Second example: in a heroic effort to query whether or not HIV neutralization could be uncoupled from antibody Fc function in mediating *in vivo* protection against HIV in a rhesus macaque model, the effectiveness of an intact (neutralizing) human IgG1 antibody was compared with the same antibody mutated and diminished in its capacity to activate complement and bind FcR (but retaining its full *in vitro* neutralization capability as well as its *in vivo* half-life). In the former situation (native antibody), eight of 9 NHP were protected from infection and disease, whereas in the latter case (Fc-dysfunctional antibody) only five of 9 animals were protected and controlled viral disease (Hessell *et al.*^2007^). The ambiguity presents at least three possible interpretations: (1) neutralization is critical, because half the animals were protected through what was argued to be a neutralization-only mechanism; (2) neutralization is insufficient, because unprotected animals had serum neutralization titers equivalent to protected animals, and diminution of Fc function ablated protective antibody function in nearly half the animals; (3) Fc–FcR interactions are critical as demonstrated in another recent paper using HIV-1-infected humanized mice (Bournazos *et al.*^2014^), but in the Hessel study polymorphism in NHP FcR confounded the results with native and modified human IgG1. Third example: broadly reactive MAbs against influenza hemagglutinin stalk—MAbs with genuine if atypical neutralizing activity *in vitro* (Tan *et al.*^2012^)—were found to be dependent upon Fc gamma receptor (FcγR) binding for the *in vivo* protection against influenza virus (DiLillo *et al.*^2014^). This contrasted with MAbs against hemagglutinin ‘head’, which were not FcγR-dependent in their *in vivo* protection. The anti-stalk MAbs induced ADCC, whereas the anti-head MAbs did not, and a variety of structure-function questions about CTAbs were revealed (*ibid*).

## CTABS IN CANCER IMMUNOTHERAPY

Decades of research, along with many MAbs licensed for clinical use against cancerous cells, underscore many of the same mechanisms by which CTAbs act against virus-infected cells (Golay and Introna 2012; Lindorfer *et al.*^2012^; Modjtahedi, Ali and Essapen 2012). For these anti-cellular mechanisms to be effective against viruses, they need not prevent viral infection at the cellular level but only diminish the burst size i.e. the number of total infectious virions produced per infected cell. The exponential result of the (presumptive) killing of virus-infected cells *in vivo* may not by itself eliminate all virus, but can clearly be sufficient to forestall disease and death while additional immune mechanisms join the fray. In this respect, CTAb may be no more or less effective than robust cytotoxic T cell responses, which are uncontroversial as mediators of antiviral immunity. As noted previously (Schmaljohn 2013), any inclination to label all such antiviral CTAb as neutralizing antibodies, whether or not they have neutralizing activity *in vitro* (simply because they are antibodies and diminish infection *in vivo*) is nonsensically akin to speaking of tumor-neutralizing Abs or virus neutralization by T cells.

## EXPERIMENTS WAITING, LOW-HANGING FRUIT

An array of clarifying experiments is possible with today's technology, and some might have been done earlier if given priority. For Ebola, but also for a wide range of viruses unconstrained by BSL4 biocontainment, it is now possible to examine in great detail the effects of Fc–FcR interactions in antiviral immunity. For a MAb of given specificity and *in vitro* function, it is relatively easy to create a family of antibodies, each with different Fc, and test their *in vivo* functions in the face of viral infection. Moreover, knockout mice of numerous types, including various FcR knockouts, are more widely available (Boesch, Brown and Ackerman 2015; Bruhns and Jönsson 2015). By manipulating Ig heavy chain (Fc), Ig glycosylation and FcR—and also by understanding allosteric communications among Fab, Fc and FcR—there is finally opportunity to explore the questions posed in the abstract: What kinds of anti-Ebola antibodies are predictably desirable in treating the afflicted, and what antibodies may account for the specific and lasting protection elicited by the more effective vaccines?

## A NOTE OF CAUTION ON SPECIES AND ALLELIC DIFFERENCES

Obviously enough, human or murine MAbs may not only elicit anti-species antibodies in NHP, but may interact in unexpected ways with FcR of mismatches species. Together with species differences in susceptibility to lethal virus infection—and differences in effector cells predominant in FcR-dependent mechanisms in a given species—such mismatches may explain circumstances in which viral neutralization is helpful but insufficient. Less obviously but becoming more clear, FcR and Fc allotypes may confound otherwise simple antibody transfer experiments, and the picture becomes far more complicated when antibody glycosylation is taken into account. Many of the complications and cautions, still unfolding, are reviewed in a recent volume (Hogarth 2015).

## CONCLUSION

Using Ebola virus as an archetype of great interest but also with many other examples cited, we have attempted to highlight the importance of antiviral defense mechanisms that involve antibodies capable of marking virus-infected cells for attack by FcR-bearing effector cells. In doing so, we set aside a longstanding term of ‘protective non-neutralizing antibodies’ in favor of cell-targeting antibodies (CTAb), acknowledging that many neutralizing antibodies are CTAb as well. This reflects an increasingly detailed understanding of the importance of Fc-dependent activities of antibodies manifested at surfaces of viral antigen-expressing cells. The purpose is not to dismiss operationally defined and useful terms like neutralization and ADCC, but to harmonize understandings wherever possible in an increasingly complex picture of antibody-mediated antiviral mechanisms that occur *in vivo*.

## Supplementary Material

Supplementary Data
