# Supplementary material for: Cell-targeting antibodies in immunity to Ebola
Source: Pathog Dis. 2016 Mar 21;74(4):ftw021. doi: 10.1093/femspd/ftw021 (PMC4835745; doi:10.1093/femspd/ftw021)
Supplement: Supplementary Data [file ftw021_supplementary_data.zip › supplement.pptx]

## Slide 1
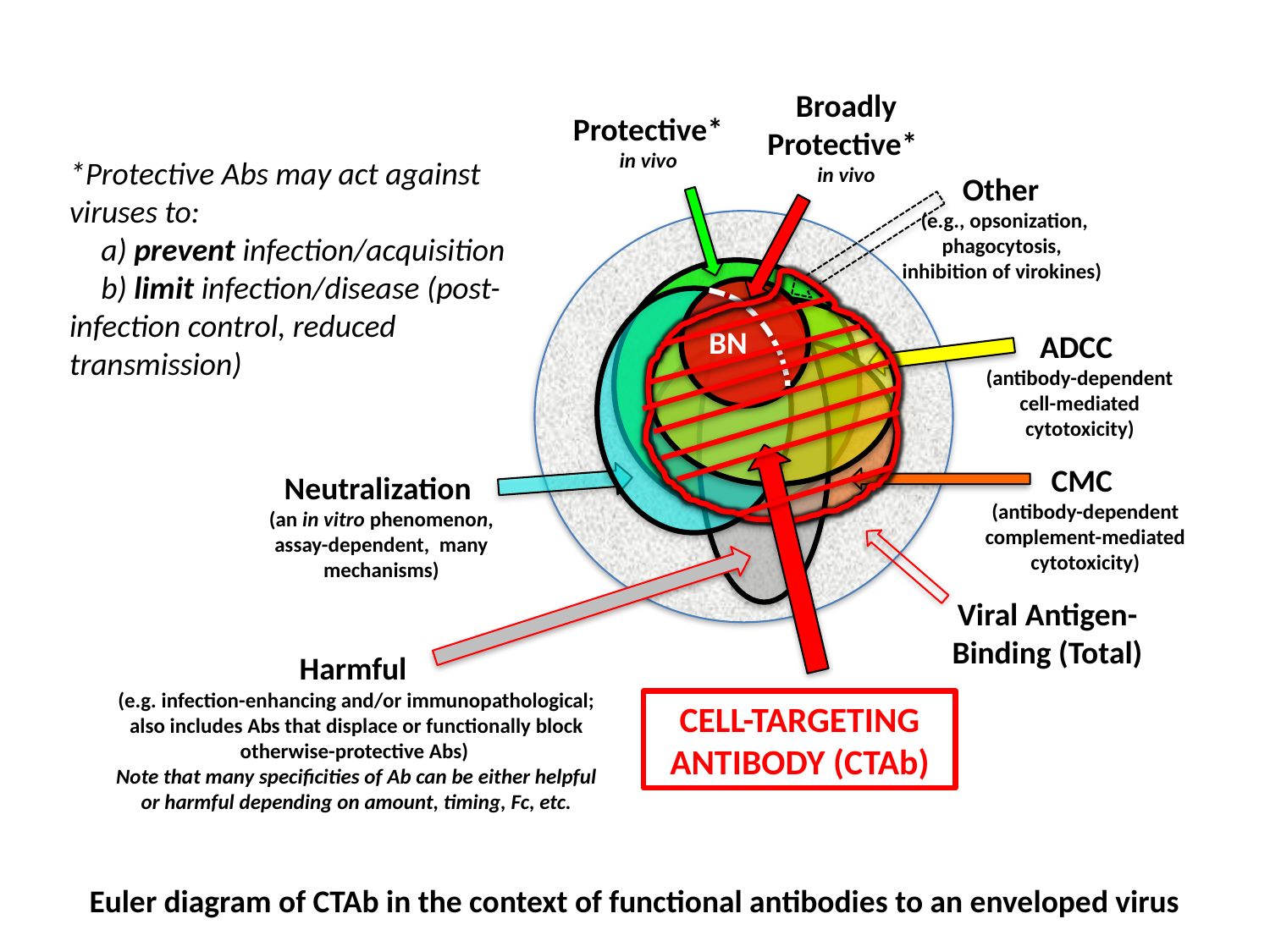

Broadly
Protective*
in vivo
Protective* in vivo
*Protective Abs may act against viruses to:
	a) prevent infection/acquisition
	b) limit infection/disease (post-infection control, reduced transmission)
Other
(e.g., opsonization, phagocytosis,
inhibition of virokines)
Viral Antigen-Binding (Total)
CELL-TARGETING ANTIBODY (CTAb)
Neutralization
(an in vitro phenomenon, assay-dependent, many mechanisms)
BN
ADCC
(antibody-dependent cell-mediated cytotoxicity)
Harmful
(e.g. infection-enhancing and/or immunopathological; also includes Abs that displace or functionally block otherwise-protective Abs)
Note that many specificities of Ab can be either helpful or harmful depending on amount, timing, Fc, etc.
CMC
(antibody-dependent complement-mediated cytotoxicity)
Euler diagram of CTAb in the context of functional antibodies to an enveloped virus
